# Association Between Magnetoencephalographic Interictal Epileptiform Discharge and Cognitive Function in Young Children With Typical Development and With Autism Spectrum Disorders

**DOI:** 10.3389/fpsyt.2018.00568

**Published:** 2018-11-19

**Authors:** Tetsu Hirosawa, Mitsuru Kikuchi, Mina Fukai, Shoryoku Hino, Tatsuru Kitamura, Kyung-Min An, Paul Sowman, Tetsuya Takahashi, Yuko Yoshimura, Yoshiaki Miyagishi, Yoshio Minabe

**Affiliations:** ^1^Department of Psychiatry and Neurobiology, Graduate School of Medical Science, Kanazawa University, Kanazawa, Japan; ^2^Department of Cognitive Science, Australian Hearing Hub, Macquarie University, Sydney, NSW, Australia; ^3^Research Center for Child Mental Development, Kanazawa University, Kanazawa, Japan; ^4^Department of Neuropsychiatry, Ishikawa Prefectural Takamatsu Hospital, Ishikawa, Japan; ^5^Health Administration Center, University of Fukui, Fukui, Japan

**Keywords:** autism spectrum disorder, magnetencephalography, epileptiform discharges, epilepsy, cognitive function

## Abstract

Electroencephalograms of individuals with autism spectrum disorders (ASD) show higher rates of interictal epileptiform discharges (IEDs), which are known to have an inverse association with cognitive function in typically developed (TD) children. Nevertheless, that phenomenon has not been investigated adequately in children with ASD. From university and affiliated hospitals, 163 TD children (84 male, 79 female, aged 32–89 months) and 107 children (85 male, 22 female, aged 36–98 months) with ASD without clinical seizure were recruited. We assessed their cognitive function using the Kaufman Assessment Battery for Children (K-ABC) and recorded 10 min of MEG. Original waveforms were visually inspected. Then a linear regression model was applied to evaluate the association between the IED frequency and level of their cognitive function. Significantly higher rates of IEDs were found in the ASD group than in the TD group. In the TD group, we found significant negative correlation between mental processing scale scores (MPS) and the IED frequency. However, for the ASD group, we found significant positive correlation between MPS scores and the IED frequency. In terms of the achievement scale, correlation was not significant in either group. Although we found a correlative rather than a causal effect, typically developed children with higher IED frequency might better be followed up carefully. Furthermore, for children with ASD without clinical seizure, clinicians might consider IEDs as less harmful than those observed in TD children.

## Introduction

Autism spectrum disorder (ASD) is a lifelong, often severely impairing neurodevelopmental syndrome characterized by impaired social cognition and communication as well as repetitive or obsessive behavior and interests (1). Its increasing prevalence (2–4), a lack of generally accepted pharmacological interventions, profound impact on quality of life, and high costs of education and care for people with ASD all combine to make ASD a public health crisis.

In addition to social impairment, most individuals with ASD have co-occurring intellectual disability (ID) (5). Results of recent studies suggest that ID in ASD might emerge as a consequence of social-communication deficits. According to this model, because social input is crucially important for normal brain development (6), poor sociality in children with ASD stunts their cognitive development by precluding them from social experience (6, 7). Supporting this view, Vianti et al. reported from their recent study that children with greater ASD severity at an initial assessment were more likely to present with poorer cognitive outcomes at later assessment, irrespective of initial cognitive level (8). Furthermore, an intervention program specifically targeting ASD symptoms in young children is known to enhance their cognitive development (9).

The association between ASD and epilepsy has been known since the first described cases (10). The prevalence of seizures in children with ASD is reportedly higher than in the general population [5–46 vs. 2–7% (11–13)]. As many as 32% of patients with epilepsy meet the diagnostic criteria of ASD (14). Moreover, even in the absence of clinical seizures, 6.7–50% of patients with ASD have IEDs shown by their electroencephalograms (EEG) (15, 16), which is much higher than the rate found among typically developing individuals: 1–4% (17, 18). Nevertheless, the clinical significance of IEDs for ASD remains unclear.

IEDs are thought to be representative of excessive neuronal activity (19). Therefore, higher IED frequency implies an excitatory shift in the equilibrium between excitatory (E) glutamatergic and inhibitory (I) gamma-amminobutyric acid (GABA) system. In fact, results of several studies suggest that the GABAergic system is disrupted in cases of ASD. For instance, genetic reports have described consistently that mutations in genes regulating GABA_A_ receptor expression occur in patients with ASD (20). Furthermore, environmental risk factors for ASD, such as exposure to maternal inflammation in prenatal life, disrupt gene expression across GABA pathways (21–23). Recently, based on such results, E/I imbalance has attracted attention as a candidate of the final “common pathways” in ASD (24).

Relation between IEDs and cognitive function in children remains unclear. In healthy individuals, most studies have found associations between the existence of IEDs and lower cognitive function (25, 26). However, for ASD, few studies have specifically examined this relation. Moreover, their results are conflicting (16, 27–30).

Those conflicting results obtained from ASD individuals might derive from methodological differences. First, the EEG duration varies among the studies. Longer EEG durations might enhance the sensitivity for detection of IEDs. Second, few studies of children with ASD have addressed the IED frequency in previous conventional studies for children with ASD (16, 28–30). Frequency of IEDs is more important than its mere existence if one considers that IEDs might affect cognitive dysfunction. Finally, most of the studies included children and adult ASD participants (14, 25–27). Because animal studies showed that IED effects on cognitive function in children might be different from the effects in adults (31), results found for inhomogeneous populations might not reflect the true relation. A noteworthy exception is a study conducted by Gillian et al. with no such limitations (27). They found no correlation between the IED frequency and cognitive dysfunction in children with ASD.

As described herein, we investigate the association between IEDs and cognitive function in children with ASD and compare it with that in typically developing children of a control group. We hypothesized that higher frequency of IEDs corresponds to lower level of cognitive function in children with ASD as well as TD children. We used magnetoencephalography (MEG) instead of EEG because it is more sensitive to epileptiform activity (32).

## Material and methods

### Participants

From Kanazawa University and affiliated hospitals, we recruited 163 typically developed children (TD) (84 male, 79 female, aged 32–89 months) and 107 children (85 male, 22 female, aged 36–98 months) with ASD. The ASD diagnosis was made according to the Diagnostic and Statistical Manual of Mental Disorders (4th edition) (DSM-IV) (1) using the Diagnostic Interview for Social and Communication Disorders (DISCO) (33) or the Autism Diagnostic Observation Schedule-Generic (ADOS-G) (34). The exclusion criteria included known blindness, hearing loss, and ID. Additionally, we excluded participants who had clinical diagnosis of any other neuropsychiatric disorder including epilepsy, and excluded participants who were receiving antiepileptic drugs. Parents agreed to the participation of children. Written informed consent was obtained before participation. The Ethics Committee of Kanazawa University Hospital approved the methods and procedures, all of which were performed in accordance with the Declaration of Helsinki.

### Assessment of cognitive function

Cognitive function of the participants was assessed using the Japanese version of the K-ABC, (35), which measures the degree of skills in various cognitive domains and which presents them on two global scales: The MPS, which measures fluid intelligence, and the Achievement Scale (ACH), which measures crystallized intelligence. These scores are provided as age-adjusted standardized scores. The scores are normalized to have mean of 100 and standard deviation of 15.

### MEG recordings

Conditions used for MEG recordings were identical to those used in our earlier study (36). MEG data were recorded using a 151-channel Superconducting Quantum Interference Device (SQUID), whole-head coaxial gradiometer MEG system for children (PQ 1151R; Yokogawa/KIT, Kanazawa, Japan) in a magnetically shielded room (Daido Steel Co., Ltd., Nagoya, Japan) installed at the MEG Center of Ricoh Co., Ltd. (Kanazawa, Japan). The custom child-sized MEG system facilitates the measurement of brain responses in young children, which would otherwise be difficult using conventional adult-sized MEG systems. The child-sized MEG system ensures that the sensors are positioned easily and effectively for the child's brain and ensures that head movements are constrained (37).

The band pass-filtered MEG data (0.16–200 Hz) were collected at a sampling rate of 1,000 Hz. During MEG recording, one staff member escorted each participant into the shielded room, which was decorated with colorful pictures of Japanese (cartoon) characters and those resembling an attractive vehicle adopted from an animation series that is popular with preschool children. During measurements, the staff member stayed in the shielded room comforting and encouraging each participant to maintain a steady body position when necessary. Parent(s)/caretaker(s) were able to observe their child during measurements through a TV monitor. During the MEG recording, the children lay supine on a bed and viewed a video program projected onto a screen (i.e., eye-open condition). The position of the head within the helmet during the MEG recording was determined by measuring the magnetic fields after passing currents through coils attached at three locations on the head surface, which served as fiduciary marks for the bilateral mastoid processes and nasion. Before recording, we prepared several video programs that were entertaining for young children. Each participant was shown a video program they had selected. Before recording, each child confirmed that the video program contents had been selected. MEG was recorded for 600 s. The time of MEG recording was between 11 a.m. and 3 p.m. No child showed a clear sign of drowsiness in terms of MEG waveforms.

### Assessment of MEG recordings

One investigator (author T.H.) reviewed the raw MEG signals (i.e., time vs. amplitude waveforms). He had been trained in EEG/MEG and epilepsy for 11 years and had extensive experience in distinguishing epileptiform discharges from other non-epileptic waveforms.

During review, T.H was blinded to the patients' names and their clinical information. To review the MEG record, the band pass filter (0.5–70 Hz) was applied. We divided all sensor pairs into eight groups (frontal/temporal/vertex/occipital region in the left and right side each). It was necessary to review all channels. T.H reviewed every group of channels by switching the montage display. He was allowed to move the records backward and forward at any time to confirm his findings. After counting of epileptiform discharges in 600 s, he reported it as a frequency per 10 s (i.e., the number of epileptiform discharges was divided by 60). Typically, this procedure required 30 min per person.

**Figure 1:**
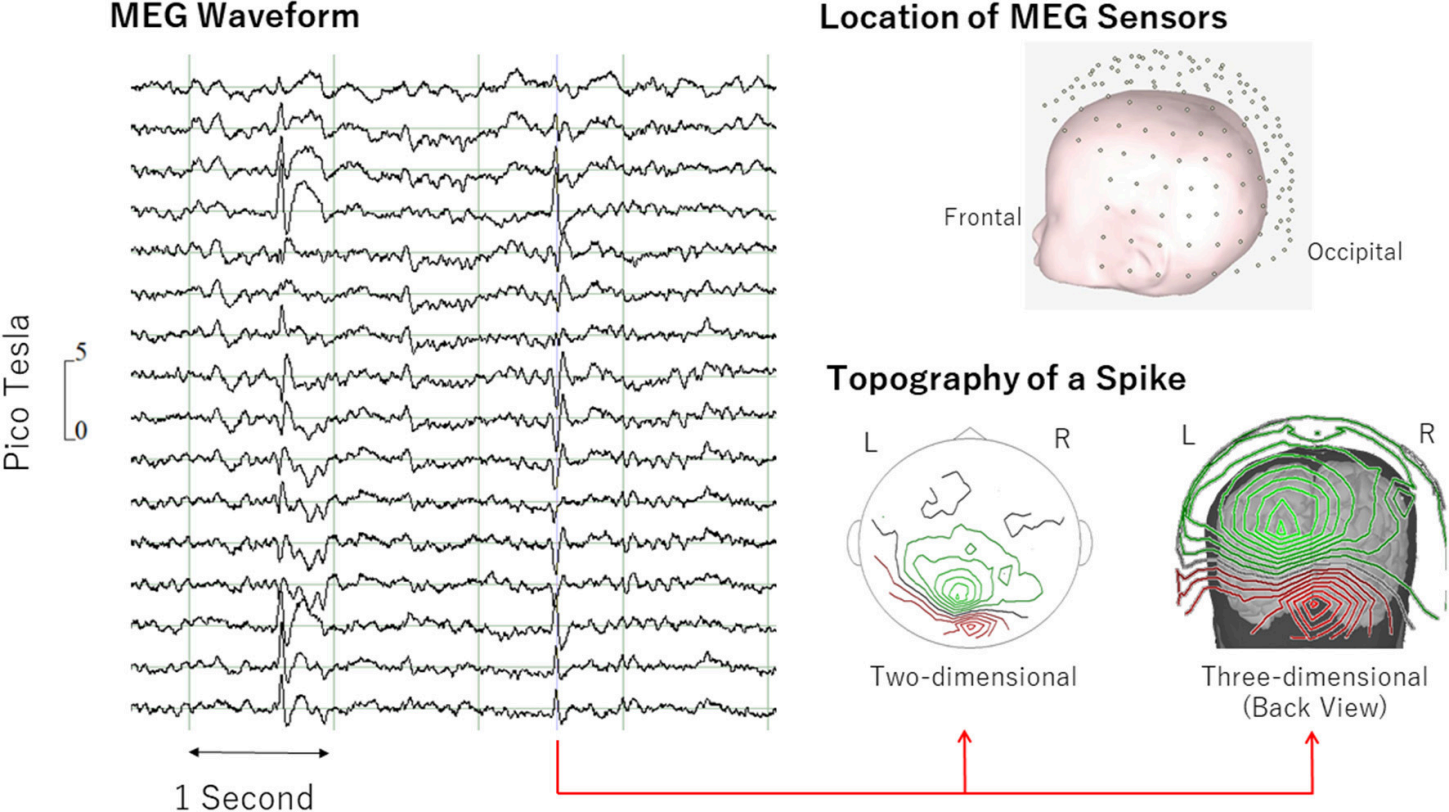
Examples of epileptiform discharge. Epileptiform discharge was defined as “sharp transient clearly different from background activity with an “epileptiform” morphology and a logical spatial distribution” (left). MEG topography (right) shows clear pattern of sink (green) and source (red). MEG, magnetoencephalography.

Epileptic discharges were detected manually by application of the same general principles recommended by the International Federation of Clinical Neurophysiology (38) and were used in standard EEG interpretation: The sharp transient is clearly different from background activity with an “epileptiform” morphology and a logical spatial distribution (e.g., Figure 1). The IED location was defined using an intermediate point of sink and source.

### Statistical analysis

Differences in population descriptors between TD and ASD were tested using Student *t*-tests for age and cognitive performance (i.e., ACH and MPS in K-ABC), and using Mann–Whitney U-tests for the IED frequency (i.e., number of IEDs per 10 s). Chi-square tests were used for analyses of the IED frequency and sex.

First, we performed a linear regression to predict MPS/ACH scores based on frequency of IEDs, disease condition (TD vs. ASD). Statistical significance was inferred for *P* < 0.05.

Before calculating a linear regression, we applied regression diagnostics to verify how well our data met the regression analysis assumptions. Specifically, we checked the following assumptions: linearity, normality, homogeneity of variance, model specification, influence and collinearity.

We examined the relation between variables using a series of scatterplots and augmented component-plus-residual plots for the variables (i.e., MPS or ACH scores and frequency of IEDs). We found no clear non-linear pattern. Here, there was no reason to check for satisfying the assumption in bivariate categorical variable because the relation is linear by definition. To check for normality in the residuals, we used kernel density plots, histograms, standardized normal probability plots, and quintile-normal plots. Based on results of those tests, we concluded that residuals were normally distributed for all models.

The graphical and the Breusch–Pagan test suggested the possible presence of heteroscedasticity in our model. To address that problem, we decided to use heteroscedasticity-robust standard errors (39).

To check the model specification error, we performed a model specification link test and a regression specification error test. Both tests gave results that were not significant for all models we employed. To check outliers, we used the added-variable plots. All data points were apparently in the range. No outlier was observed. Multicollinearity was tested using the variance inflation factor (VIF). No variable had VIF >10 or 1/VIF < 0.10 for any model.

If significant interaction involving the disease condition was found, then a linear regression model was applied within ASD and within TD to ascertain the association between the IED frequency and level of cognitive function (i.e., ACH and MPS in K-ABC) controlling for age and sex. Again, according to the regression diagnostics, we decided to use heteroscedasticity-robust standard errors. Statistical significance was inferred for *P* < 0.025 (Bonferroni correction for multiple comparisons was applied).

In the regression diagnostics, violation was found only for homoscedasticity. Therefore, we decided to use heteroscedasticity-robust standard errors.

Then, because of a possible influence of sex and age on MPS/ACH (31, 40, 41), we entered those in the regression model to control for the confounding effects. Against the ACH and MPS scores in K-ABC (i.e., dependent variable), the model included age, sex, IED frequency, child condition (TD vs. ASD), and interaction between the IED frequency and condition. Because the regression diagnostics showed heteroscedasticity, we decided to use heteroscedasticity-robust standard errors. Statistical significance was inferred for *P* < 0.05.

If significant interaction involving the disease condition was found, then we applied a linear regression model within ASD and within TD to ascertain the association between the IED frequency and cognitive function (i.e., ACH and MPS in K-ABC) controlling for age and sex. Again, according to the regression diagnostics, we decided to use heteroscedasticity-robust standard errors. In these models, statistical significance was inferred for *P* < 0.025 (Bonferroni correction for multiple comparisons was applied).

All statistical analyses were performed using software (Stata ver. 15.0; Stata Corp. College Station, TX, USA).

## Results

**Figure 2:**
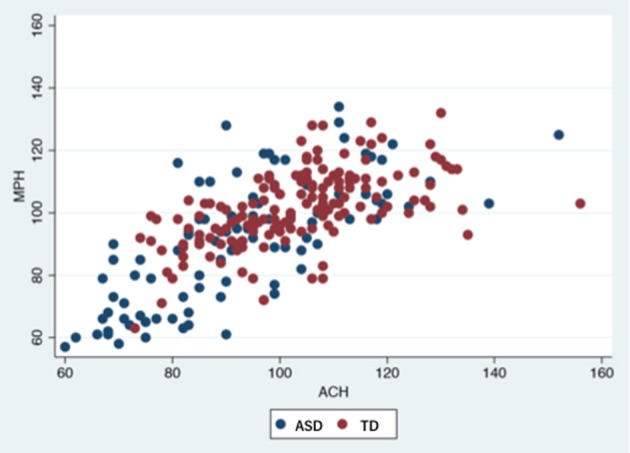
Participants' performance in K-ABC.ASD, autism spectrum disorder; TD, typically developed controls; K-ABC, Kaufman assessment battery for children; MPS, mental processing scale; ACH, achievement scale.

Statistical analyses were conducted after excluding 22 children (7 TD, 15 ASD) who were unable to complete the K-ABC or MEG recording. Actually, 12 of these children (5 TD, 7 ASD) had unreadable MEG recordings because of motion artifacts, noise artifacts or environmental interference. None of the participants received medication. Significant differences were found in age, sex, cognitive performance, prevalence, and the IED frequency between TD and ASD groups. Tables 1, 2 and Figure 2 present results.

**Table 1:** Characteristics of participants.

	**TD**	**ASD**	**χ^2^**	***t***	***z***	***p***	**Effect size**
*n*	156	92					
Gender (male/female)*	81/75	72/20	17.0			< 0.05	0.26
Months^#^	56.5 (11.8)	65.5 (12.6)		5.7		< 0.05	0.74
Prevalence of IEDs (Negative/positive)*	140/16	73/19	5.2			< 0.05	0.14
IED frequency (per 10 s)^$^	0.06 (0.02)	0.10 (0.04)			2.2	< 0.05	0.14
**K-ABC scores**							
Mental processing scale^#^	101.7 (12.0)	91.9 (19.8)		−4.8		< 0.05	0.63
Achievement scale^#^	103.1 (14.7)	93.8 (18.6)		−4.3		< 0.05	0.83

*Numbers are mean (standard deviation) or counts*.

*Effect sizes were provided by Cramer's V for chi-square test, Cohen's d for Student' t-test and z value divided by square root of sample size for Mann–Whitney U-test*.

* *Chi-square test*.

# *Student t-test*.

$ *Mann–Whitney U-test*.

*ASD, autism spectrum disorder; TD, typically developed controls; K-ABC, Kaufman assessment battery for children*.

**Table 2:** Distribution of IEDs.

	**TD**		**ASD**	
	**R**	**L**	**R**	**L**
Frontal lobe	2	1	4	3
Temporal lobe	0	4	3	4
Parietal lobe	3	2	5	4
Occipital lobe	3	4	1	4
Multiple focus	2		6	

*Numbers are counts*.

*The location of IED was defined using an intermediate point of sink and source*.

*ASD, autism spectrum disorder; R, right; L, left; TD, typically developed controls*.

### Association between the IED frequency and level of cognitive function

For MPS scores, significant main effects were found for the condition [*t*_(244)_ = −4.6, *p* < 0.05] and the IED frequency [*t*_(244)_ = −5.3, *p* < 0.05]. Significant interaction effects were also found between the IED frequency and disease condition [*t*_(244)_ = 4.8, *p* < 0.05]. The relation between the IED frequency and MPS scores differed depending on the disease condition.

For ACH scores, a significant main effect was found for the disease condition [*t*_(244)_ = −4.1, *p* < 0.05]. No other factors were found to be significant. The results are presented in Table 3.

**Table 3:** Association between IED frequency and level of cognitive function.

**variable**	**Coeff**.	**SE**	***t***	**95% CI**	***p***
**MENTAL PROCESSING SCALE**					
Condition (ASD = 1/TD = 0)	−10.8	2.3	−4.6	−15.4 to −6.2	< 0.05
IED frequency (/10 s)	−7.7	1.5	−5.3	−10.6 to −4.9	< 0.05
Condition × IED frequency	14.0	2.9	4.8	8.2 to 19.7	< 0.05
**ACHIEVEMENT SCALE**					
Condition (ASD = 1/TD = 0)	−9.6	2.3	4.1	−14.2 to −4.9	< 0.05
IED frequency (/10 s)	−2.1	3.0	−0.7	−8.1 to 3.8	0.48
Condition × IED frequency	4.0	4.9	0.8	−5.6 to 13.7	0.41

*Coef., regression coefficient; SE, robust standard error; CI, confidence interval; ASD, autism spectrum disorder; TD, typically developed controls*.

### Different association between the IED frequency and level of cognitive function in each group

To elucidate the association between the IED frequency and the level of cognitive function, a linear regression model with robust variance estimation was applied within each group.

In the TD group, for MPS scores, a significant main effect of the IED frequency [*t*_(154)_ = −5.31, *p* < 0.025] was found. Higher IED frequency predicted lower MPS scores. For ACH, the main effect was not found to be significant.

In the ASD group, for MPS scores, a significant main effect of the IED frequency on the MPS [*t*_(90)_ = 2.47, *p* < 0.025] was found. Higher IED frequency predicted higher MPS scores. For the ACH, the effect was not found to be significant. The results are presented in Table 4.

**Table 4:** Association between IED frequency and level of cognitive function in each group.

**TD**					
**Variable**	**Coeff**.	**SE**	***t***	**95% CI**	***p***
**Mental processing scale**					
IED frequency (/10 s)	−7.7	1.4	−5.3	−10.6 to −4.9	< 0.025
**Achievement scale**					
IED frequency (/10 s)	−2.1	3.0	−0.7	−8.0 to 3.8	0.48
**ASD**					
**Variable**	**Coeff**.	**SE**	***t***	**95% CI**	***p***
**Mental processing scale**					
IED frequency (/10 s)	6.2	2.5	2.5	1.2 to 11.2	< 0.025
**Achievement scale**					
IED frequency (/10 s)	1.9	3.9	0.5	−5.8 to 9.6	0.63

*Coeff., regression coefficient; SE, robust standard error; CI, confidence interval; ASD, autism spectrum disorder; TD, typically developed controls*.

### Association between the IED frequency and level of cognitive function controlling for age and sex

For MPS scores, significant main effects were found for the condition [*t*_(242)_ = −4.77, *p* < 0.05] and the IED frequency [*t*_(242)_ = −4.89, *p* < 0.05]. Significant interaction effects were also found between the IED frequency and disease condition [*t*_(242)_ = 4.45 *p* < 0.05]. No other factor was found to be significant. The relation between the IED frequency and MPS scores differed depending on the disease condition after controlling for age and sex.

For ACH scores, a significant main effect was found for the disease condition [*t*_(242)_ = −3.14, *p* < 0.05]. ASD children had lower ACH scores compared to TD children. No other factor was found to be significant. The results are presented in Table 5.

**Table 5:** Association between IED frequency and level of cognitive function controlling for age and sex.

**Variable**	**Coeff**.	**SE**	***t***	**95% CI**	***p***
**MENTAL PROCESSING SCALE**					
Condition (ASD = 1/TD = 0)	−12.2	2.6	−4.8	−17.2 to −7.1	< 0.05
IED frequency (/10 s)	−8.0	1.6	−4.9	−11.3 to −4.8	< 0.05
Condition × IED frequency	14.3	3.2	4.4	8.0 to 20.6	< 0.05
Age (months)	0.15	0.8	1.93	−0.0 to 0.3	0.05
Sex (male = 1/female = 0)	0	2	0	−3.9 to 3.8	0.99 <
**ACHIEVEMENT SCALE**					
Condition (ASD = 1/TD = 0)	−8.8	2.8	−3.1	−14.3 to −3.3	< 0.05
IED frequency (/10 s)	−1.83	3.1	−0.6	−7.9 to 4.2	0.55
Condition × IED frequency	3.3	4.9	0.7	−6.3 to 13.0	0.49
Age (months)	0	0.1	0	−0.2 to 0.2	0.96
Sex (male = 1/female = 0)	−2.7	2.3	−1.2	−7.3 to 1.8	0.25

*Coeff., regression coefficient; SE, robust standard error; CI, confidence interval; ASD, autism spectrum disorder; TD, typically developed controls*.

### Different association between the IED frequency and level of cognitive function controlling for age and sex in each group

To elucidate the effects of the IED frequency on cognitive function, a linear regression model with robust variance estimation was applied within each group.

In the TD group, for MPS scores, a significant main effect of the IED frequency [*t*_(152)_ = −4.77, *p* < 0.025] was found. No other factor was found to be significant. Higher IED frequency predicted lower MPS scores after controlling for age and sex. For ACH, no factor was found to be significant.

In the ASD group, for MPS scores, a significant main effect of the IED frequency on the MPS [*t*_(88)_ = 2.35, *p* < 0.025) was found. No other factor was found to be significant. Higher IED frequency predicted higher MPS scores after controlling for age and sex. For the ACH, no factor was found to be significant. The results are presented in Table 6.

**Table 6:** Association between IED frequency and level of cognitive function controlling for age and sex in each condition.

**TD**					
**variable**	**Coeff**.	**SE**	***t***	**95% CI**	***P***
**Mental processing scale**					
IED frequency (/10 s)	−7.7	1.6	−4.8	−10.8 to −4.5	< 0.025
Age (months)	0.1	0.1	1.2	−0.1 to 0.2	0.25
Sex (male = 1/female = 0)	−2.6	1.9	−1.4	−6.2 to 1.2	0.18
**Achievement scale**					
IED frequency (/10 s)	−1.8	3.1	−0.6	−7.9 to 4.3	0.57
Age (months)	0	0.1	−0.2	−0.2 to 0.2	0.88
Sex (male = 1/female = 0)	−3.2	2.4	−1.4	−8.0 to 1.4	0.17
**ASD**					
**variable**	**Coeff**.	**SE**	***t***	**95% CI**	***p***
**Mental processing scale**					
IED frequency (/10 s)	7.2	3.0	2.35	1.1 to 13.3	< 0.025
Age (months)	0.2	0.2	1.36	−0.1 to 0.5	0.18
Sex (male = 1/female = 0)	6.6	4.8	1.37	−2.9 to 16.1	0.17
**Achievement scale**					
IED frequency (/10 s)	1.7	4	0.4	−6.2 to 9.6	0.68
Age (months)	0	0.2	0.1	−0.4 to 0.4	0.91
Sex (male = 1/female = 0)	−1.4	5.5	−0.3	−12.2 to 9.5	0.78

*Coeff., regression coefficient; SE, robust standard error; CI, confidence interval; ASD, autism spectrum disorder; TD, typically developed controls*.

## Discussion

This report is the first of a study providing evidence demonstrating that the association between the IED frequency and level of cognitive function in children with ASD differ from those in a control group of TD children. First, using a linear regression model, we demonstrated that the association between the IED frequency and MPS scores differed depending on the child condition. This association remained significant after controlling for age and sex. Then, *post-hoc* analysis showed that higher IED frequency predicted lower MPS scores for TD children. However, in the ASD group, higher IED frequency predicted higher MPS scores. These associations also remained significant after controlling for age and sex.

In our study, 10.3% of TD children and 20.7% of children with ASD had at least one IED in their MEG recordings. For TD children, the rate was slightly higher than reported previously. However, for children with ASD, reported rates vary: 6.7–61%. (18) This discrepancy might be explained by differences in the type of EEG used in those studies because, for example, prolonged sleep EEG reportedly has higher sensitivity for IEDs than routine EEG has (28). In fact, studies using routine EEGs found similar rates [e.g., 5.7% (42), 18% (43)] to those we observed. Studies using longer durations of EEGs found much higher rates. For example, 61% was reported by (44). Comparing the duration of routine EEG recording (typically about 60 min) and the much shorter duration of our MEG recording (600 s), one might infer that MEG has higher sensitivity for IEDs, perhaps because of inherent differences between MEG and EEG.

We found a higher prevalence of IEDs in children with ASD than in TD children. IEDs are presumed to represent excessive neuronal activity. Therefore, the existence of IEDs can imply a local excitatory shift in the E/I balance. Supporting this view, studies using proton magnetic resonance spectroscopy ([^1^H]MRS) have shown higher local brain glutamate and lower GABA level in patients with ASD than in healthy controls (45, 46). The higher frequency of IEDs suggest an ongoing regional E/I imbalance in the brains of children with ASD.

The MPS scores represent a person's fluid intelligence, a purely general ability to discriminate and perceive relations between any fundaments (47). In terms of MPS scores, we found a significant interaction effect between the IED frequency and child condition. Results of *post-hoc* analysis showed that higher IED frequency predicted lower MPS scores for TD children. Considering the inverse association between IEDs and level of fluid intelligence for TD children, IEDs can be pathogenetic in this population. Although we found a correlative rather than a causal effect, typically developed children with higher IED frequency must be followed up carefully. In some cases with concurrent cognitive impairment, antiepileptic treatment might be considered (48).

However, for children with ASD, *post-hoc* analysis revealed that higher IED frequency predicted higher MPS scores. Therefore, our results did not support the pathogenicity of IEDs in children with ASD. For children with ASD, clinicians might consider the IEDs as less harmful than those observed in TD children. Rather, a higher frequency of IEDs would correspond to better fluid intelligence. In addition, based on our results, the nature of IEDs might differ in children with ASD from that in TD. Possibly, considering higher prevalence of IED in children with ASD, pathology of ASD can play a role in this phenomenon. For example, E/I imbalance (observed as IEDs) in children with ASD can be an epiphenomenon or compensatory changes to the underlying cause of ASD. In support of this hypothesis, it is noteworthy that Gerhard et al. reported that despite an excitatory shift in E/I balance in the occipital region in ASD participants, the neurons in that region were not severely damaged (45). Furthermore, recent results of results of studies suggest that underlying causes of ASD can have positive effects on their fluid intelligence (49, 50).

The ACH scores represent a person's crystallized intelligence. We found significant main effects of disease condition on ACH scores. Particularly, children with ASD had significantly lower ACH scores than TD children had. Consistent with these results, studies cited above also suggest that the underlying cause of ASD adversely affects their crystallized intelligence (49, 50). Nevertheless, no correlation was found between the IED frequency and the ACH scores of either group. Possible explanations include the lack of statistical power and the existence of potential confounders. For example, because crystallized intelligence relies on acquired knowledge, it might be susceptible to the educational environment. To examine the IED effects on ACH, further study must be undertaken including environmental information such as socioeconomic status, and presence or lack of early childhood education.

Limitations of our study are as follows. First, most of the children with ASD examined in this study were high-functioning. Consequently, the findings of this study might not be applicable to children with “Kanner's autism.” However, although we excluded children with known neuropsychiatric disorders other than ASD, the participants still possibly had comorbid disorders. For example, some developmental disorders (e.g., attention deficit/hyperactivity disorder, and learning disability) are difficult to detect at this age. Further studies that consider comorbid disorders using larger sample sizes must be conducted because these comorbidities might affect cognitive performance. Second, the participants were recruited from a small region. Considering environmental risk factors for ASD, our results should be generalized with caution. Third, we assigned only one investigator (T.H.) to review all the MEG recordings. He was instructed to look for transients and to make an “epileptic” or “non-epileptic” decision for every suspicious waveform. Although we had a rigorous definition of epileptic discharge, in truth, sometimes it was difficult to make a decision. It was preferable to employ another rater to cross-check the decisions. Fourth, we were not able to ascertain the precise location of the source of the magnetic field as we could if we used anatomical images such as those gained from MRI. We judged that most of the subjects in our study were unable to endure the examination of conventional magnetic resonance imaging (MRI). Future studies using child-friendly, open-type MRI devices and/or brain models that match the individual brain are necessary to reduce uncertainty in source level estimation. Fifth, we recorded MEG for only 10 min. We would have had higher sensitivity for IEDs if we had recorded MEG for at least 20 min as The American Clinical Neurophysiology Society recommends (51). In addition, presumably because of this short recording time, no child examined for this study showed any clear sign of drowsiness in terms of MEG waveform. Importantly, however, some autistic features are known to occur in the context of sleep IEDs (e.g., the Landau–Kleffner syndrome and electrical status epilepticus in slow wave sleep) (52). To elucidate the relation between IEDs and cognitive function further, future studies must particularly address the difference between the effect of sleep and awake IEDs on cognitive function.

In conclusion, this report described the negative association between the IED frequency and MPS scores in TD children. Although we found a correlative rather than a causal effect, typically developed children with higher IED frequency must be followed up carefully. We also reported a positive association between the IED frequency and MPS scores in children with ASD. For children with ASD without clinical seizure, clinicians might consider the IEDs as less harmful than those observed in TD children.

Our results demonstrate the possibility that E/I imbalance in children with ASD is epiphenomenal or compensatory, rather than causal in ASD. However, drawing such a hard conclusion based purely on our results is inappropriate. In TD children, to clarify a causal relation, studies must be conducted to prove (or disprove) the effectiveness of antiepileptic treatment on various aspects of cognitive function. In ASD children, in view of the developmental aspect of the disease, a longitudinal follow-up study considering the relation between IEDs and cognitive function should be conducted. Change in cognitive function over the course of time in relation to IEDs might provide additional information related to the nature of IEDs in this population. For such clinical trials, given the negative correlation between the IED frequency and cognitive function observed in TD children, not only should the existence of IEDs be assessed; the IED frequency should be assessed as well.

## Author contributions

TH and MK designed the study and wrote the protocol. MK and YMin supervised the research. TH wrote the first draft of the manuscript. PS, TT, and TK revised the manuscript and advised colleagues on statistical methods and composition of the manuscript. TH, MF, and SH conducted statistical analyses. YY and K-MA recruited participants. YMiy and YY recorded MEG. All authors contributed to and approved the final manuscript.

### Conflict of interest statement

The authors declare that the research was conducted in the absence of any commercial or financial relationships that could be construed as a potential conflict of interest.
